# QuickProt: A bioinformatics and visualization tool for DIA and PRM mass spectrometry-based proteomics datasets

**DOI:** 10.1101/2025.03.24.645047

**Published:** 2025-03-28

**Authors:** Omar Arias-Gaguancela, Carmen Palii, Mehar Un Nissa, Marjorie Brand, Jeff Ranish

**Affiliations:** 1 Institute for Systems Biology, Seattle, WA, USA; 2 Wisconsin Blood Cancer Research Institute, Department of Cell and Regenerative Biology, Wisconsin Institutes for Medical Research, University of Wisconsin-Madison, Madison, WI, USA

**Keywords:** Data-independent acquisition, erythropoiesis, liquid chromatography-tandem mass spectrometry, mass spectrometry, parallel reaction monitoring, proteomics, QuickProt, stable isotope dilution

## Abstract

Mass spectrometry (MS)-based proteomics focuses on identifying and quantifying peptides and proteins in biological samples. Processing of MS-derived raw data, including deconvolution, alignment, and peptide-protein prediction, has been achieved through various software platforms. However, the downstream analysis, including quality control, visualizations, and interpretation of proteomics results remains challenging due to the lack of integrated tools to facilitate the analyses. To address this challenge, we developed QuickProt, a series of Python-based Google Colab notebooks for analyzing data-independent acquisition (DIA) and parallel reaction monitoring (PRM) proteomics datasets. These pipelines are designed so that users with no coding expertise can utilize the tool. Furthermore, as open-source code, QuickProt notebooks can be customized and incorporated into existing workflows. As proof of concept, we applied QuickProt to analyze in-house DIA and stable isotope dilution (SID)-PRM MS proteomics datasets from a time-course study of human erythropoiesis. The analysis resulted in annotated tables and publication-ready figures revealing a dynamic rearrangement of the proteome during erythroid differentiation, with the abundance of proteins linked to gene regulation, metabolic, and chromatin remodeling pathways increasing early in erythropoiesis. Altogether, these tools aim to automate and streamline DIA and PRM-MS proteomics data analysis, making it more efficient and less time-consuming.

## Introduction

Over the last few decades, mass spectrometry (MS)-based proteomics has emerged as one of the most dominant fields for protein profiling [1,2]. Advances in high-resolution MS instruments and computational tools have enabled researchers to characterize large-scale proteomes across various biological systems, with applications ranging from cell state transitions to structural biology and drug design [3,4]. Data-independent acquisition (DIA) is an untargeted MS technique used in proteomics analysis for the identification and relative quantification of peptides and proteins [5,6]. Unlike conventional approaches such as data-dependent acquisition (DDA), where precursor peptide ions are typically selected for fragmentation based on their intensities, DIA uses a series of predefined mass-to-charge (m/z) windows to select and fragment co-eluting precursor ions simultaneously [5,7,8]. This allows for the unbiased and often more reproducible identification of peptides and proteins, making it ideal for discovery proteomics experiments [9,10].

Parallel reaction monitoring (PRM) is a targeted MS strategy in which the mass spectrometer is programmed to analyze a predefined set of m/z values corresponding to peptides of interest [11,12]. By focusing on a predetermined set of target peptides, the technique can achieve low detection limits and high reproducibility levels. Compared to wide-window DIA, the resulting fragment ion chromatograms from PRM are less complex, simplifying their deconvolution and downstream processing [13,14]. Combined with stable isotope dilution (SID), PRM allows absolute quantification of peptides and proteins [15].

Although many software tools have been developed to identify and quantify peptide/proteins from raw LC-MS/MS data, such as DIA-NN [16], Spectronaut, OpenMS [17] and EncyclopeDIA [18], only a few offer the tools needed for quality control, data visualization, and statistical analysis (e.g., MSstats) [19] among the proteomes of experimental groups. While some tools, like Perseus [20], do provide some of these services, users are often limited to settings, pre-established by the developer, restricting the number and types of possible customizations. Here, we present QuickProt, a series of seven Python-based Google Colab notebooks dedicated to the data mining and visualization of DIA and PRM MS-proteomics datasets (Figure S1 and Table S1).

### QuickProt development and implementation

**Figure 1:**
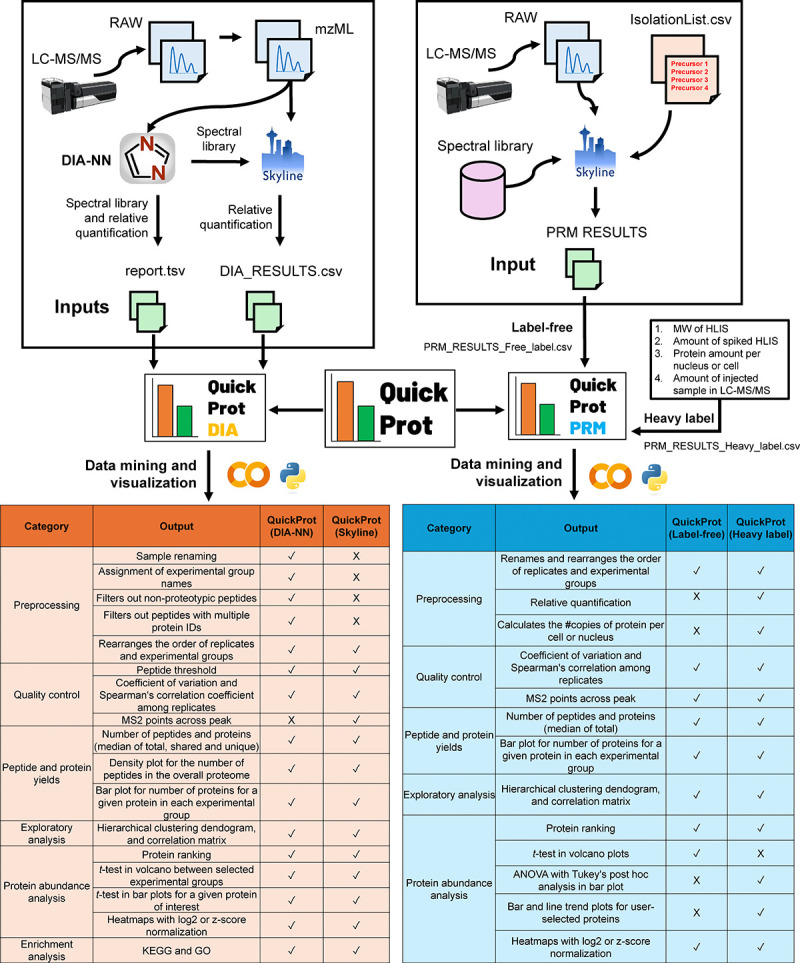
Overview of QuickProt Google Colab notebook tool for DIA and PRM analysis. In the left panel, the QuickProt-DIA workflow is represented. LC-MS/MS data is exported in RAW file format and subsequently converted to mzML format. Next, mzML files are imported into DIA-NN software for peptide and protein identification and relative quantification. Alternatively, mzML files can be imported into Skyline for quantification while using the DIA-NN spectral library for identification. Skyline or DIA-NN tables from either workflow can be analyzed in the Python-based QuickProt-DIA module composed of QuickProt-DIA (DIA-NN) or QuickProt (Skyline) notebooks. Both generate annotated tables and figures that fall into the following categories: preprocessing (e.g., sample filtering), quality control (e.g., coefficient of variation), peptide and protein yields, exploratory analysis (e.g., hierarchical clustering dendrogram), protein abundance (e.g., heatmap visualization), and enrichment analysis (e.g., KEGG). In the right panel, the QuickProt-PRM module workflow is represented. LC-MS/MS data in RAW file format is imported into Skyline alongside an isolation list (IsolationList.csv) and a selected spectral library. Following Skyline analysis, the output table ‘PRM_RESULTS_Free_label.csv’ or ‘PRM_RESULTS_Heaby_label.csv’ is imported into QuickProt-PRM for analysis. QuickProt (Label-free) notebook outputs are generated automatically, whereas, for heavy-label experiments, QuickProt (Heavy-label) notebook requires additional information (e.g., amount of spiked HLIS) for the generation of absolute quantities. The lower left and right panels show tables with the contents produced for each notebook. Abbreviations: LC-MS/MS: liquid chromatography-tandem mass spectrometry; mzML: Mass Spectrometry Markup Language; MW: molecular weight; HLIS: heavy labeled internal standard; KEGG: Kyoto Encyclopedia of Genes and Genomes; GO: gene ontology.

The QuickProt tool is designed to analyze DIA and PRM data through QuickProt-DIA and QuickProt-PRM modules, respectively (Figure 1, Figure S1). It enables proteomics data analysis to be more efficient and less time-consuming by automating several tasks. Users with no coding expertise can easily utilize these tools. The user only needs to provide a single input table generated from DIA-NN [16] or Skyline [21] software, two widely-used, freely available algorithms used for the initial analysis of DIA and PRM analysis, respectively. By simply clicking on the run button, users can execute all the codes in the notebooks. All outputs from these notebooks, including tables and figures, will be saved in dedicated folders. The resulting spreadsheet tables will be saved in a folder called ‘TABLES’ while the generated figures will be saved as TIFF images at a resolution of 300 dpi in the folder “PLOTS” (Table S1). Furthermore, as open-source code, these tools can be modified for other bioinformatics workflows depending on the needs of the investigator. All notebooks can be easily accessed at https://github.com/OmarArias-Gaguancela/QuickProt, where all of the modules have descriptions and links to their respective notebooks or pipelines.

### QuickProt-DIA module overview

The QuickProt-DIA module includes two pipelines, depending on whether the input file is obtained from DIA-NN or Skyline. (Figure 1, Figures S1–S5). QuickProt-DIA (DIA-NN) pipeline uses the output table from DIA-NN, namely ‘report.tsv’, as an input file that contains peptides and proteins identified through their neural network-based spectral library and quantified by a MaxLFQ-like algorithm [16]. On the other hand, QuickProt-DIA (Skyline) pipeline uses a table from Skyline named ‘DIA_RESULTS.csv’ for processing. In QuickProt-DIA (Skyline), we used the DIA-NN spectral library for identification and Skyline to quantify peptides and proteins. Depending on the input table (DIA-NN or Skyline), the QuickProt-DIA module produces outputs that fall into the following categories: preprocessing, quality control, analysis of peptide and protein yields, exploratory analysis, protein abundance analysis, and gene enrichment analysis (Figure 1).

#### Preprocessing:

During the preprocessing stage, the user can rename the samples and add experimental group names to the replicates of a given condition (Figure S2), a function not currently available in DIA-NN but provided in Skyline. In the case of QuickProt-DIA (DIA-NN), we filtered out non-proteotypic peptides and peptides that were assigned multiple Protein IDs in the DIA-NN prediction. Notably, both QuickProt-DIA pipelines allow customization of the layout of the samples and experimental groups. We incorporated an optional control point to filter out proteins that do not meet a specified unique peptide threshold (Figure S2). This feature is particularly beneficial for removing proteins that are identified by only a single peptide, which could result in false positives and/or unreliable quantification [22]. Implementing this type of quality control is recommended in discovery MS proteomics experiments [22].

#### Quality Control:

For quality control purposes, we included a section to generate a coefficient of variation (CV) plot and correlation plot that displays Spearman’s correlation coefficient and data distribution among the replicates/samples of a given experimental group. For QuickProt-DIA (Skyline), we added a section for plotting the distribution of MS points across the chromatographic peaks for each experimental group. Since DIA quantification is generally based on MS2 points, we used MS2 points as the default choice. However, this can be adjusted to MS1, depending on the objective of the analysis (Figure S3). We highly recommend first running these sections of the notebook, as these outputs help to assess the reproducibility and technical variability in the experiment.

#### Peptide and Protein Yield:

Next, QuickProt-DIA generates annotated tables and plots for the total number of peptides and proteins found in a given experiment (Figure S3). Additionally, it provides a comprehensive breakdown of the number of shared proteins between experimental groups and the number of unique proteins in each experimental group. These results are concurrently extracted into annotated CSV tables, which are deposited in a subfolder called SHARED_UNIQUE_PROTEINS within the TABLES folder, allowing easy access for the user (Table S1). These pipelines also allow the user to display a density plot for the number of peptides and calculate the median number of peptides per protein in the dataset. Additionally, users can enter the name of a specific protein or gene of interest to automatically visualize the number of peptides detected for that protein in the dataset.

#### Exploratory Analysis:

In the exploratory analysis, the user can display hierarchical clustering dendrogram and correlation matrix plots to identify groupings and connectivity relationships between the experimental groups tested in the experiment (Figure S3).

#### Protein Abundance:

For protein abundance visualization (Figure S4), we introduce a protein ranking tool where the user can array the proteins identified in each experimental group and rank them based on their estimated abundance levels. We provide a dedicated section for visualizing relative protein abundances through a volcano plot. For this analysis, a *t*-test is performed. Users can compare the fold change (FC) and *P*-values of the proteins between two experimental groups. To make it more interactive and user-friendly, we added drop-down menus allowing users to select *P*-values (0.05 to 0.001) and FC thresholds (0.5 to 10) that fit the experiment’s needs. These values are then converted to −log10 and log2 scales for plotting. Accordingly, a list of the total number of proteins analyzed is generated as an output table, along with lists of only upregulated or downregulated proteins in a subfolder called VOLCANO_PLOT_VALUES within the TABLES folder (Table S1). We went a step further by creating a section (Figure S4) that allows the user to visualize individual protein abundances across experimental samples by simply typing the name of the protein or gene of interest and then clicking a “Generate Plot” button to yield a bar plot with its respective statistical analysis. The user selects the reference experimental group against which the statistical analysis will be performed. The bars of the plot will be automatically assigned an asterisk for statistically significant differences, or the letters ‘n.s.’ will be added on top of the bar when no statistical differences in protein abundances are found between the samples of interest.

The user has the option to plot the abundances of all quantified proteins in each experimental group or sample via clustering heatmap visualization (Figure S4). We added a section where the user can input a list of proteins or genes of interest to be displayed in a heatmap. In this case, neither imputation nor clustering is conducted. Missing values are depicted with the letters ‘n.d,’ whereas the normalized values for the abundances of the selected proteins are shown on the log2 scale. Alternatively in another section, we used the ‘IterativeImputer’ tool from the Scikit-learn machine learning library [23], which uses an iterative approach to handle missing values, and then this data is used to display a clustering heatmap comparing the proteomes among experimental groups.

#### Enrichment Analysis:

We incorporated the Kyoto Encyclopedia of Genes and Genomes (KEGG) and Gene Ontology (GO) tools in the enrichment analysis section of the QuickProt-DIA pipelines to identify biological pathways, processes, components, and functions that are enriched in a dataset. Hence, QuickProt-DIA facilitates KEGG and GO analyses on DIA datasets to provide a systems view of the biological processes that are affected in an experimental group, which can help prioritize proteins for potential follow-up experiments. In these pipelines, the user can choose whether to perform the analysis on the list of total proteins of a given experimental group, the total number of proteins compared in the volcano plot, or exclusively on upregulated or downregulated proteins derived from the same volcano plot analysis (Figure S5).

### QuickProt-PRM module overview

QuickProt-PRM module comprises two pipelines, namely, QuickProt-PRM (Label-free) and QuickProt-PRM (Heavy label), for processing of PRM data from Skyline through input tables titled ‘PRM_RESULTS_Free_label.csv’ or ‘PRM_RESULTS_Heavy_label.csv’, respectively (Figure 1, Figures S6–S10). Similar to the DIA module, QuickProt-PRM also produces outputs including categories such as preprocessing, quality control, peptide and protein yields, exploratory analysis, and protein abundance analysis. During the preprocessing step, customization of the sample and experimental group layout is provided for both notebooks (Figures S6–S7). QuickProt-PRM (Label-free) is dedicated to relative abundance quantification, whereas QuickProt-PRM (Heavy label) aims to calculate the number of molecules of a given protein in a sample using spiked-in isotopically heavy labeled internal standard (HLIS) proteins or peptides. These values can then be converted to copies per cell or organelle (e.g., nucleus) with the appropriate conversion factors. To accomplish such absolute calculations, the user must provide the molecular weight of the spiked HLIS, the known amount of protein per nucleus or cell used in the experiment, and the amount of sample injected into the LC-MS/MS (Figure S7). CV, correlation, and MS2 point plots can be generated during the quality control stage. The user can plot the number of quantified peptides for a given protein/gene, and view the total number of peptides and proteins quantified. Exploratory analysis through hierarchical clustering dendrogram and correlation matrix is also provided (Figure S8).

Like in QuickProt-DIA, QuickProt-PRM (Label-free) allows the user to visualize the estimated relative abundance of proteins via protein ranking, volcano, bar plots, and heatmaps (Figure S9). For QuickProt-PRM (Heavy label), absolute quantities of a protein (e.g., #copies/nucleus or #copies/cell) can be visualized through bar and/or line trend plots. We provide a section where users can select a given protein of interest and assess the statistical differences in abundance among experimental groups. In addition, one of the advantages of using PRM proteomics with HLIS is the ability to compare stoichiometric changes between different proteins. QuickProt-PRM leverages this by enabling an option to display and assess the statistical differences (Figure S10) between different proteins in a given experimental group. In both types of comparisons, ANOVA with Tukey’s post-hoc statistical tests are performed, and the results are displayed automatically in the bar plots. Indeed, we used the compact letter display to automatically depict significant differences among multiple groups. Finally, QuickProt-PRM allows users to display the abundance of all the targeted proteins for all experimental groups or by sample via clustering heatmaps. There is also an option to specifically display certain proteins of interest in the heatmap.

Lastly, we introduce two more modules, named QuickProt-PepSeq and QuickProt-ID Search. The first module is designed to evaluate the number of peptides identified in a specific amino acid sequence (e.g., domain) from a DIA dataset (Figure S11). This module is compatible with inputs from DIA-NN or Skyline reports processed using QuickProt-DIA, specifically ‘report_updated.csv’ or ‘DIA_RESULTS_UPDATED.csv’, respectively. In both notebooks, users simply need to input the sequence of interest and click the run button to produce an Excel spreadsheet (Table S1) with annotated tabs, displaying the number of peptide matches and their respective sequences by sample and experimental group. A bar plot of the number of peptide matches is also displayed (Figure S11). QuickProt-ID Search maps protein IDs in the UniProt database to a list of gene names provided by the user (Figure S12). This is especially helpful in cases where the investigator deals with thousands of genes.

### DIA dataset and analysis using QuickProt-DIA

#### DIA dataset:

**Figure 2:**
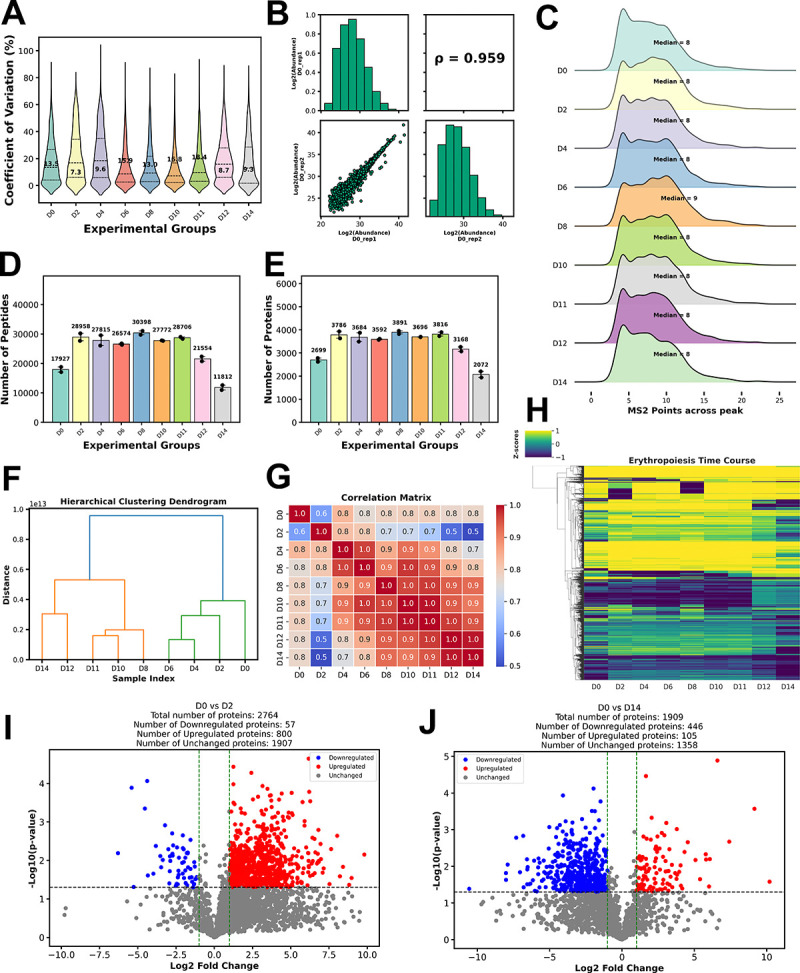
Comprehensive analysis of erythropoiesis time-course DIA proteomics data via QuickProt-DIA (Skyline) notebook. Samples on days 0, 2, 4, 6, 8, 10, 11, 12, and 14 (D0–14) during erythroid differentiation were collected for DIA discovery proteomics analysis. A) Coefficient of variation, with median values depicted inside each violin plot. B) Spearman’s correlation coefficient (ρ) and data distribution plots among replicates for day 0. C) Distribution of MS2 data points across the chromatographic peak, with median values depicted for the proteomes of each experimental group. D) Number of peptides identified in each experimental group, with median values depicted on top of each bar graph. E) Number of proteins identified in each experimental group, with median values depicted on top of each bar graph. Data represent the median ± SD of two biological replicates. F) Hierarchical clustering dendrogram. G) Correlation matrix among experimental groups. H) Clustering heatmap. I) Volcano plot for differential expression between D0 and D2. J), and D0 and D14. For I) and J), a *P*-value ≤ 0.05 and an FC (fold-change) threshold of > |2| were chosen for the analyses.

To demonstrate the performance of QuickProt-DIA, we analyzed an in-house generated DIA dataset. We employed a well-characterized *ex vivo* culture system method in which cord blood-derived human multipotent hematopoietic stem and progenitor cells (HSPCs) were induced to differentiate along the erythroid lineage [24,25] (Method S1). Samples were collected on days 0, 2, 4, 6, 8, 10, 11, 12, and 14, representing sequential stages of erythropoiesis, thus yielding cell lineages from HSPCs (day 0) to polychromatophilic and orthochromatic erythroblasts (day 14). Nuclear extracts were prepared from the cells (Method S1) and used to generate peptide samples for LC-MS/MS analysis on an Orbitrap Eclipse mass spectrometer operated in DIA mode (Method S2–S4; Table S2 and S4). During further processing, we used DIA-NN to generate a spectral library for peptide and protein identification due to its high sensitivity and processing speed. Peptide-to-protein quantification was achieved with Skyline. Chromatograms were extracted, and qualitative analysis of each product ion peak was performed, providing an additional safeguard to enhance data reliability. The quantitative information was exported from Skyline which was then processed using the QuickProt-DIA (Skyline) pipeline (Figure 2).

#### DIA analysis results:

For this analysis, the minimum peptide threshold per protein was set at two for all samples in the dataset. Then, QuickProt-DIA was used to evaluate the quality and reproducibility of the DIA data. The CVs varied from 7.3% to 18.4% in the experimental groups tested (Figure 2A). Since these values were below the recommended 20% threshold [26–28], this suggests acceptable biological variability among the replicates. The correlation plot for Day 0 replicates shows a Spearman’s rank correlation coefficient of 0.959 (Figure 2B), and similar results were obtained for the other days of the time course (Figure S13). We also examined the distribution of MS2 points across chromatographic peaks; in this experiment, the median ranged from 8 to 9 MS2 points (Figure 2C). This range has been reported to be optimal for appropriately representing the peptide peak shape for accurate quantification [29]. Lastly, we assessed the median number of peptides per protein in each experimental group and found a median value of 4 to 5 for peptides per protein (Figure S14A). These metrics suggest that the LC-MS/MS method used was able to meet certain quality control requirements, ensuring robust quantitative accuracy for the experiment.

Regarding peptide and protein identification during the time course study, the total number of peptides varied from 11,812 to 30,398 (Figure 2D), yielding 2,072 to 3,891 proteins (Figure 2E), respectively. Notably, the core proteome representing the number of proteins shared between all experimental groups consisted of 1,813 identified proteins. The number of shared proteins between different samples varied from 96 to 1,958 (Figure S14B). Day 8 had the highest number of unique proteins (75), and Days 6 and 12 had the lowest number of unique proteins (3–4) (Figure S14C).

Inspection of the outputs from the exploratory analysis tools revealed a marked grouping at certain stages of erythropoiesis progression. Hierarchical clustering identified two major branches. The first branch comprised two sub-branches: one with day 0, and the other with days 2, 4, and 6. By contrast, the second branch contained two major sub-branches; one comprised of days 12 and 14, and the other of days 8, 10, and 11 (Figure 2F). In the correlation matrix, days 0 and 2 had the lowest levels of correlation compared to the other days of the time course, particularly when compared to days 12 and 14 (Figure 2G). This suggests timely and dynamic changes from early to late stages of erythropoiesis. This pattern is reminiscent of what was observed in the clustering analysis. When performing a clustering heatmap, the protein expression profiles for each day were readily visualized, and differentially expressed proteins could be easily detected. (Figure 2H). Given the important role of chromatin-modifying complexes in transcriptional regulation during erythropoiesis through modulation of chromatin accessibility [30], we used QuickProt-DIA to inspect the expression profiles of subunits of a few chromatin-modifying complexes [31], including the following: BAF (BRG1- or BRM-associated factor), ISWI (Imitation Switch), NuRD (Nucleosome Remodeling and Deacetylase), INO80 (Inositol requiring 80), SAGA (Spt-Ada-Gcn5 Acetyltransferase), ATAC (Ada Two-A Containing), SRCAP (Snf2-related CREBBP Activator Protein), and ATR-X (Alpha Thalassemia/Mental Retardation Syndrome X-linked) (Figure S15). QuickProt-DIA generated heatmaps that allowed easy visualization of the expression patterns and relative abundances for the members of these complexes over the time course. Interestingly, the abundances of several members of the BAF, e.g., ARID1A, and ISWI, e.g., SMARCA5 complexes, peaked at days 2 and 8, implying a requirement for these subunits at these specific time points during erythropoiesis (Figure S15).

Next, we used QuickProt-DIA to evaluate the protein abundance rankings for proteomes on each day of erythropoiesis (Figure S16). Notably, AHNAK was consistently the most abundant protein at all-time points.

Supported by the initial evidence from the exploratory analysis, we hypothesized that the number of upregulated proteins would increase significantly from the initial starting point on day 0 throughout certain time points during erythropoiesis progression. To validate this hypothesis, we used the volcano plot tool (Figure 2I–J; Figure S17) in the QuickProt-DIA (Skyline) pipeline and performed a statistical analysis of the proteomes of day 0 compared to each subsequent day. Similar to the day 0 vs. day 2 comparison (Figure 2I), when day 0 was compared to days 4–11, approximately 93% of the significantly changed proteins were upregulated, while the rest were downregulated. Notably, after day 11, the number of upregulated proteins declined, continuing until day 14 (Figure 2I and J; Figure S17). On day 14, out of the 551 significantly changed proteins, around 81% were downregulated, and 19% were upregulated (Figure 2J). Consistent with these findings, when inspecting individual proteins (e.g., ARID1A and SMARCC2), we observed a dramatic spike on day 2 and a decrease on days 12 and 14 compared to day 0 (Figure S18). These data reveal the dynamics of the proteome during erythropoiesis, and suggest that specific proteins may need to be upregulated in the early stages, whereas certain proteins may require downregulation in the later stages for proper differentiation

Lastly, we used the KEGG and GO tools in the QuickProt-DIA notebook to evaluate the biological role of proteins identified during different stages of erythropoiesis (Figures S19–S26). KEGG and GO data showed no major differences when inspecting the total number of proteins (Figure S19, S22–S24). However, focusing on significantly upregulated or downregulated proteins revealed some interesting findings. For instance, in the day 0 vs. day 2 comparison, upregulated proteins at day 2 were enriched in processes like spliceosome activity, primary metabolism (e.g., Fatty acid metabolism), and ATP-dependent chromatin remodeling (Figure S20A). The enrichment of proteins involved in chromatin remodeling at day 2 is consistent with the notion of priming at the early stages of development when the chromatin is relatively open [32], but transcription is still low. Conversely, in the day 0 vs. day 14 comparison, pathways associated with phagosome vesicle production were most enriched (Figure S20B) at day 14, likely linked to the extrusion of nuclei in the late stages of erythropoiesis [33–35]. By contrast, downregulated proteins in the day 0 vs. day 2 comparison exclusively affected pyruvate and glycolysis metabolism, while the day 0 vs. day 14 comparison showed downregulation in pathways related to spliceosome activity, DNA replication, nucleocytoplasmic transport, carbon metabolism, among others (Figure S21). This analysis indicates that certain pathways enriched on day 2 are downregulated at later stages of erythroid development, suggesting a timely and dynamic regulation during erythropoiesis.

We assessed molecular function, cellular components, and biological processes in the GO analysis (Figures S22–S26). For example, the upregulated proteins on day 2 showed enrichment for translation, ribosome biogenesis, nucleic acid, and protein binding processes (Figure S25). At day 14, cellular and protein localization, transport and binding, and ATP-related processes were prominent (Figure S25). Conversely, downregulated proteins on day 2 favored processes such as hemoglobin binding and cellular detoxification, while on day 14, protein and mRNA binding and processing, and chromosome reorganization were affected (Figure S26). Altogether, the QuickProt DIA (Skyline) pipeline efficiently provides an overview of the biological processes underlying erythroid differentiation, establishing an initial framework for evaluating complex proteomic datasets to extract meaningful biological insights.

### PRM dataset and analysis using QuickProt-PRM

#### PRM dataset:

**Figure 3:**
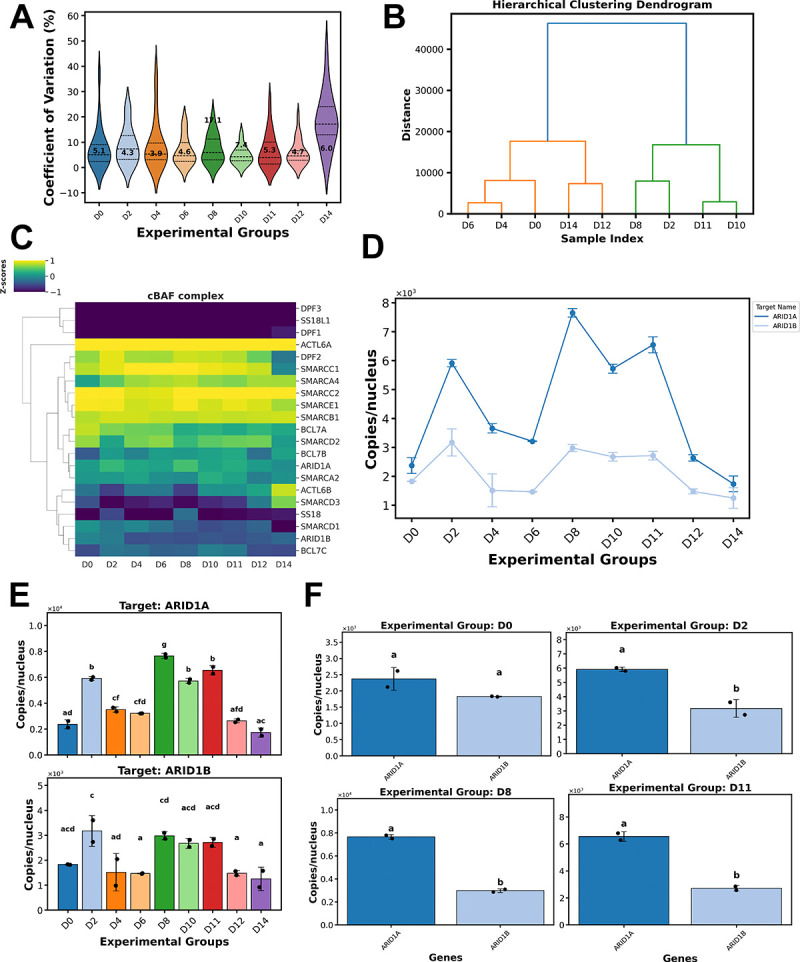
Targeted analysis of cBAF proteins during erythropoiesis via QuickProt-PRM (Heavy-label) notebook. Erythroid samples were spiked with heavily labeled concatamers (QconCATs) for absolute quantification (#copies/nucleus) of 21 cBAF proteins during erythroid differentiation via PRM proteomics. A) Coefficient of variation, with median values depicted inside each violin plot. B) Hierarchical clustering dendrogram. C) Clustering heatmap for proteins in the cBAF complex. D) Trend line plot for ARID1 paralogues, ARID1A and ARID1B. E) Bar plots for the absolute abundance of ARID1A and ARID1B, each plotted independently throughout the time course. F) Stoichiometric evaluation between ARID1A and ARID1B was conducted at four time points (D0, D2, D8, and D11). Data represent the median ± SD of two biological replicates. A compact letter display is automatically assigned on top of every bar. Different letters denote significant differences (*P* ≤ 0.05) by ANOVA with Tukey’s post-hoc test.

The mammalian SWI/SNF complex, also known as the BAF complex, is an ATP-dependent chromatin remodeling complex that plays important roles in gene regulation during cell differentiation [36–42]. This remodeler is organized into three distinct configurations: canonical-BAF (cBAF), polybromo-associated-BAF (PBAF), and non-canonical-BAF (ncBAF) [36,43]. Each complex comprises 12–15 subunits, with some subunits shared between different complexes, and paralogous subunits present for several of them. Mutations in protein components of BAF have been linked to numerous forms of cancer, neurodevelopmental disorders, and defective erythropoiesis [36,37,40,44,45]. An analysis of BAF subunit stoichiometry during erythropoiesis would provide insights into the dynamic regulation of the complex during erythropoiesis and can add to the existing knowledge on its role in chromatin remodeling and transcription [40]. Towards this aim, we determined the absolute abundances of 21 subunits (including paralogues) of the cBAF complex (all subunits except for ACTB) by SID-PRM mass spectrometry during the time course of erythropoiesis. This dataset was then analyzed using QuickProt-PRM (Heavy label) for easy visualization and interpretation. For this data generation, we created heavily labeled concatemer proteins (QconCATs) [46,47] containing up to 4 peptides for each cBAF subunit. Known amounts of these QconCATs were then spiked into the nuclear extract fractions followed by enzymatic digestion (Method S2–3; Table S3–S7). Peptides obtained after co-digestion were cleaned, and analyzed by PRM-MS to acquire targeted spectral information for the cBAF subunits (Method S3–4; Table S3–S4). Raw data (Table S4) was then processed in Skyline, where, after chromatogram extraction, each product ion peak was qualitatively monitored to ensure the reliability of the corresponding quantitative information. After analysis and refinement, the values were exported and analyzed using the QuickProt-PRM (Heavy label) pipeline (Figure 3).

#### PRM analysis results:

Quality control analysis revealed CVs ranging from 4.3–17.1% (Figure 3A). A Spearman’s rank correlation coefficient of approximately 0.9 among the samples (Figure S27) and a median of 28–32 MS2 points across chromatographic peaks (Figure S28A) was observed. These data indicate excellent reproducibility among the replicates from each experimental group. Exploratory analysis revealed two major groupings; the first comprised days 0, 4, 6, 12, and 14, while the second included days 2, 8, 10, and 11 (Figure 3B). Inspection of the correlation matrix showed high-level similarity among days, except for day 14, which had the lowest level of correlation compared to the others (Figure S28B).

Employing the peptide and protein yield option in QuickProt PRM (Heavy label), a total median peptide yield of 50–54 was detected for up to 21 cBAF proteins (Figure S29A–B). Peptide distribution showed a median of 2–3 peptides per protein detected (Figure S29C). Heatmap clustering and protein abundance ranking analysis revealed that among the 21 proteins quantified, ACTL6A was consistently the most abundant (42203 copies), whereas DPF1, DPF3, and SSL18L1 were the least abundant (137 copies) (Figure 3C; Figure S30; Table S8). Further, we inspected the relative stoichiometry of all cBAF paralogues during the erythroid time course (Figure 3D; Figure S31). For example, ARID1 paralogues, namely ARID1A and ARID1B, showed a similar number of copies per nucleus at day 0 (2,371 and 1,828 copies, respectively; *P*>0.05). Then, rapid and significant spikes in abundance were noticed on days 2, 8, and 11, followed by a decline until day 14 for both paralogues (Figure 3D–E). Noticeably, ARID1A yielded more copies per nucleus than ARID1B at most points of the time course (average difference of 2,763 copies on days 2–12; *P*≤0.05) (Figure 3D, F). Discordant expression levels between paralogues were also observed for ACTL6A over ACTL6B, DPF2 over DPF1 or DPF3, SMARCA4 over SMARCA2, and to a lesser extent in BCL7A over BCL7B and BCL7C, and SS18 over SS18L1 (Figure S31). Interestingly, SMARCD2 had a major spike exclusively on day 11, then dramatically decreased until day 14. Although SMARCD2 was mostly elevated relative to the other paralogues (SMARCD1 and SMARCD3), it is important to note a shift in relative abundance on day 14, where SMARCD3 was higher than the other two paralogues (Figure S31). The observed changes in the relative stoichiometry for these paralogues during the time course could indicate subunit switching [48,49] in cBAF or PBAF during erythroid development.

Unlike the other paralogues where the higher expression of one paralogue over another is evident, SMARCC1 and SMARCC2 yielded similar copy numbers throughout several data points, with some exceptions (e.g., day 14) (Figure S31). Lastly, to complete the analysis of cBAF members, we also analyzed the abundance trend of SMARCE1 (no paralogue) and found abundance spikes on days 2, 8, and 11 (Figure S31).

The data mining and visualization tools from the QuickProt-PRM notebook allowed us to efficiently assess the absolute quantities of members of the cBAF complex, highlighting their expression characteristics during erythropoiesis. This framework will be used in follow-up experiments to target specific cBAF subunits for knockdown or overexpression experiments to test the functional importance of the observed abundance measurements during erythroid differentiation.

## Conclusions and Outlook

QuickProt provides tools carefully designed to analyze and visualize data from DIA and PRM MS-proteomics experiments. In this article, we demonstrated the utility of the developed tools using in-house generated DIA and PRM datasets, offering insights into the biological processes underlying erythropoiesis. QuickProt offers comprehensive solutions beyond DIA data analysis (QuickProt-DIA). We provide notebooks for the analysis of PRM data (QuickProt-PRM), supporting both label-free and heavy-label experiments for relative and absolute quantification, respectively. Moreover, we have incorporated two more tools; QuickProt-PepSeq to report the number of unique peptide matches to a user-defined protein sequence in DIA datasets, and QuickProt-ID Search to retrieve UniProt protein IDs from gene names. We leveraged the accessibility of Google Colab notebooks to build our Python-based code within them. This makes it more user-friendly and versatile compared to other tools in the field, which typically require some coding skills. Furthermore, as an open-source platform, users can modify and customize the tools to suit the needs of specific projects.

## Supplementary Material

Supplement 1

Supplement 2

Supplement 3

## Data Availability

The LC-MS/MS raw data, spectral libraries, and the input files processed in QuickProt have been deposited in ProteomeXchange [PXD060333] and can be accessed also via Panorama Public [https://panoramaweb.org/QuickProt_datasets.url]. The QuickProt notebooks were deposited in GitHub (https://github.com/OmarArias-Gaguancela/QuickProt).

## References

[1] Shuken S.R., An Introduction to Mass Spectrometry-Based Proteomics. J. Proteome Res. 2023, 22, 2151–2171. 37260118 10.1021/acs.jproteome.2c00838

[2] Aebersold R., Mann M., Mass spectrometry-based proteomics. Nature 2003, 422, 198–207. 12634793 10.1038/nature01511

[3] Guerrera I.C., Kleiner O., Application of mass spectrometry in proteomics. Biosci. Rep. 2005, 25, 71–93. 16222421 10.1007/s10540-005-2849-x

[4] Boys E.L., Liu J., Robinson P.J., Reddel R.R., Clinical applications of mass spectrometry-based proteomics in cancer: Where are we? PROTEOMICS 2023, 23, 2200238. 10.1002/pmic.202200238 35968695

[5] Fröhlich K., Brombacher E., Fahrner M., Vogele D., , Benchmarking of analysis strategies for data-independent acquisition proteomics using a large-scale dataset comprising inter-patient heterogeneity. Nat. Commun. 2022, 13, 2622. 35551187 10.1038/s41467-022-30094-0 PMC9098472

[6] Doerr A., DIA mass spectrometry. Nat. Methods 2015, 12, 35–35.

[7] Fröhlich K., Fahrner M., Brombacher E., Seredynska A., , Data-Independent Acquisition: A Milestone and Prospect in Clinical Mass Spectrometry–Based Proteomics. Mol. Cell. Proteomics 2024, 23, 100800. 38880244 10.1016/j.mcpro.2024.100800 PMC11380018

[8] Willems P., Fels U., Staes A., Gevaert K., Van Damme P., Use of Hybrid Data-Dependent and -Independent Acquisition Spectral Libraries Empowers Dual-Proteome Profiling. J. Proteome Res. 2021, 20, 1165–1177. 33467856 10.1021/acs.jproteome.0c00350 PMC7871992

[9] Page M.J., Amess B., Rohlff C., Stubberfield C., Parekh R., Proteomics: a major new technology for the drug discovery process. Drug Discov. Today 1999, 4, 55–62. 10234157 10.1016/s1359-6446(98)01291-4

[10] Espejo C., Lyons B., Woods G.M., Wilson R., in:, Greening DW, Simpson RJ (Eds.), SerumPlasma Proteomics Methods Protoc., Springer US, New York, NY 2023, pp. 127–152.

[11] Park J., Oh H.J., Han D., Wang J.I., , Parallel Reaction Monitoring-Mass Spectrometry (PRM-MS)-Based Targeted Proteomic Surrogates for Intrinsic Subtypes in Breast Cancer: Comparative Analysis with Immunohistochemical Phenotypes. J. Proteome Res. 2020, 19, 2643–2653. 31755719 10.1021/acs.jproteome.9b00490

[12] Peterson A.C., Russell J.D., Bailey D.J., Westphall M.S., Coon J.J., Parallel Reaction Monitoring for High Resolution and High Mass Accuracy Quantitative, Targeted Proteomics*. Mol. Cell. Proteomics 2012, 11, 1475–1488. 22865924 10.1074/mcp.O112.020131 PMC3494192

[13] Urisman A., Levin R.S., Gordan J.D., Webber J.T., , An Optimized Chromatographic Strategy for Multiplexing In Parallel Reaction Monitoring Mass Spectrometry: Insights from Quantitation of Activated Kinases*. Mol. Cell. Proteomics 2017, 16, 265–277. 27940637 10.1074/mcp.M116.058172 PMC5294213

[14] Peckner R., Myers S.A., Jacome A.S.V., Egertson J.D., , Specter: linear deconvolution for targeted analysis of data-independent acquisition mass spectrometry proteomics. Nat. Methods 2018, 15, 371–378. 29608554 10.1038/nmeth.4643 PMC5924490

[15] Gillespie M.A., Palii C.G., Sanchez-Taltavull D., Shannon P., , Absolute Quantification of Transcription Factors Reveals Principles of Gene Regulation in Erythropoiesis. Mol. Cell 2020, 78, 960–974.e11. 32330456 10.1016/j.molcel.2020.03.031 PMC7344268

[16] Demichev V., Messner C.B., Vernardis S.I., Lilley K.S., Ralser M., DIA-NN: neural networks and interference correction enable deep proteome coverage in high throughput. Nat. Methods 2020, 17, 41–44. 31768060 10.1038/s41592-019-0638-x PMC6949130

[17] Röst H.L., Sachsenberg T., Aiche S., Bielow C., , OpenMS: a flexible open-source software platform for mass spectrometry data analysis. Nat. Methods 2016, 13, 741–748. 27575624 10.1038/nmeth.3959

[18] Searle B.C., Pino L.K., Egertson J.D., Ting Y.S., , Chromatogram libraries improve peptide detection and quantification by data independent acquisition mass spectrometry. Nat. Commun. 2018, 9, 5128. 30510204 10.1038/s41467-018-07454-w PMC6277451

[19] Kohler D., Staniak M., Tsai T.-H., Huang T., , MSstats Version 4.0: Statistical Analyses of Quantitative Mass Spectrometry-Based Proteomic Experiments with Chromatography-Based Quantification at Scale. J. Proteome Res. 2023, 22, 1466. 37018319 10.1021/acs.jproteome.2c00834 PMC10629259

[20] Tyanova S., Temu T., Sinitcyn P., Carlson A., , The Perseus computational platform for comprehensive analysis of (prote)omics data. Nat. Methods 2016, 13, 731–740. 27348712 10.1038/nmeth.3901

[21] MacLean B., Tomazela D.M., Shulman N., Chambers M., , Skyline: an open source document editor for creating and analyzing targeted proteomics experiments. Bioinforma. Oxf. Engl. 2010, 26, 966–968. 10.1093/bioinformatics/btq054 PMC2844992 20147306

[22] Li Y.F., Arnold R.J., Tang H., Radivojac P., The importance of peptide detectability for protein identification, quantification, and experiment design in MS/MS proteomics. J. Proteome Res. 2010, 9, 6288–6297. 21067214 10.1021/pr1005586 PMC3006185

[23] Pedregosa F., Varoquaux G., Gramfort A., Michel V., , Scikit-learn: Machine Learning in Python. J Mach Learn Res 2011, 12, 2825–2830.

[24] Giarratana M.-C., Kobari L., Lapillonne H., Chalmers D., , Ex vivo generation of fully mature human red blood cells from hematopoietic stem cells. Nat. Biotechnol. 2005, 23, 69–74. 15619619 10.1038/nbt1047

[25] Palii C.G., Cheng Q., Gillespie M.A., Shannon P., , Single-Cell Proteomics Reveal that Quantitative Changes in Co-expressed Lineage-Specific Transcription Factors Determine Cell Fate. Cell Stem Cell 2019, 24, 812–820.e5. 30880026 10.1016/j.stem.2019.02.006 PMC6886472

[26] Cho K.-C., Oh S., Wang Y., Rosenthal L.S., , Evaluation of the sensitivity and reproducibility of targeted proteomic analysis using data independent acquisition for serum and cerebrospinal fluid proteins. J. Proteome Res. 2021, 20, 4284–4291. 34384221 10.1021/acs.jproteome.1c00238 PMC8631582

[27] Brenes A.J., Calculating and Reporting Coefficients of Variation for DIA-Based Proteomics. J. Proteome Res. 2024, 23, 5274–5278. 39573822 10.1021/acs.jproteome.4c00461 PMC11629372

[28] Andersen L.-A.C., Palstrøm N.B., Diederichsen A., Lindholt J.S., , Determining Plasma Protein Variation Parameters as a Prerequisite for Biomarker Studies—A TMT-Based LC-MSMS Proteome Investigation. Proteomes 2021, 9, 47. 34941812 10.3390/proteomes9040047 PMC8707687

[29] Pino L.K., Just S.C., MacCoss M.J., Searle B.C., Acquiring and Analyzing Data Independent Acquisition Proteomics Experiments without Spectrum Libraries. Mol. Cell. Proteomics MCP 2020, 19, 1088–1103. 32312845 10.1074/mcp.P119.001913 PMC7338082

[30] Ludwig L.S., Lareau C.A., Bao E.L., Nandakumar S.K., , Transcriptional States and Chromatin Accessibility Underlying Human Erythropoiesis. Cell Rep. 2019, 27, 3228–3240.e7. 31189107 10.1016/j.celrep.2019.05.046 PMC6579117

[31] Gourisankar S., Krokhotin A., Wenderski W., Crabtree G.R., Context-specific functions of chromatin remodellers in development and disease. Nat. Rev. Genet. 2024, 25, 340–361. 38001317 10.1038/s41576-023-00666-x PMC11867214

[32] Gaspar-Maia A., Alajem A., Meshorer E., Ramalho-Santos M., Open chromatin in pluripotency and reprogramming. Nat. Rev. Mol. Cell Biol. 2011, 12, 36–47. 21179060 10.1038/nrm3036 PMC3891572

[33] Chasis J.A., Mohandas N., Erythroblastic islands: niches for erythropoiesis. Blood 2008, 112, 470–478. 18650462 10.1182/blood-2008-03-077883 PMC2481536

[34] Klei T.R.L., Meinderts S.M., van den Berg T.K., van Bruggen R., From the Cradle to the Grave: The Role of Macrophages in Erythropoiesis and Erythrophagocytosis. Front. Immunol. 2017, 8, 73. 28210260 10.3389/fimmu.2017.00073 PMC5288342

[35] Qiu L.-B., Dickson H., Hajibagheri M.A.N., Crocker P.R., Extruded Erythroblast Nuclei Are Bound and Phagocytosed by a Novel Macrophage Receptor. Blood 1995, 85, 1630–1639. 7888682

[36] Mashtalir N., Suzuki H., Farrell D.P., Sankar A., , A Structural Model of the Endogenous Human BAF Complex Informs Disease Mechanisms. Cell 2020, 183, 802–817.e24. 33053319 10.1016/j.cell.2020.09.051 PMC7717177

[37] Michel B.C., D’Avino A.R., Cassel S.H., Mashtalir N., , A non-canonical SWI/SNF complex is a synthetic lethal target in cancers driven by BAF complex perturbation. Nat. Cell Biol. 2018, 20, 1410–1420. 30397315 10.1038/s41556-018-0221-1 PMC6698386

[38] Singhal N., Graumann J., Wu G., Araúzo-Bravo M.J., , Chromatin-Remodeling Components of the BAF Complex Facilitate Reprogramming. Cell 2010, 141, 943–955. 20550931 10.1016/j.cell.2010.04.037

[39] Xiao M., Kondo S., Nomura M., Kato S., , BRD9 determines the cell fate of hematopoietic stem cells by regulating chromatin state. Nat. Commun. 2023, 14, 8372. 38102116 10.1038/s41467-023-44081-6 PMC10724271

[40] Alfert A., Moreno N., Kerl K., The BAF complex in development and disease. Epigenetics Chromatin 2019, 12, 19. 30898143 10.1186/s13072-019-0264-y PMC6427853

[41] Wu J., Fan C., Kabir A.U., Krchma K., , Baf155 controls hematopoietic differentiation and regeneration through chromatin priming. Cell Rep. 2024, 43, 114558. 39088321 10.1016/j.celrep.2024.114558 PMC11465209

[42] Park J., Kirkland J.G., The role of the polybromo-associated BAF complex in development. Biochem. Cell Biol. Biochim. Biol. Cell. 2025, 103, 1–8. 10.1139/bcb-2024-0224 PMC11752563 39541575

[43] Varga J., Kube M., Luck K., Schick S., The BAF chromatin remodeling complexes: structure, function, and synthetic lethalities. Biochem. Soc. Trans. 2021, 49, 1489–1503. 34431497 10.1042/BST20190960

[44] Boulay G., Sandoval G.J., Riggi N., Iyer S., , Cancer-Specific Retargeting of BAF Complexes by a Prion-like Domain. Cell 2017, 171, 163–178.e19. 28844694 10.1016/j.cell.2017.07.036 PMC6791823

[45] Kadoch C., Crabtree G.R., Mammalian SWI/SNF chromatin remodeling complexes and cancer: Mechanistic insights gained from human genomics. Sci. Adv. 2015, 1, e1500447. 26601204 10.1126/sciadv.1500447 PMC4640607

[46] Pratt J.M., Simpson D.M., Doherty M.K., Rivers J., , Multiplexed absolute quantification for proteomics using concatenated signature peptides encoded by QconCAT genes. Nat. Protoc. 2006, 1, 1029–1043. 17406340 10.1038/nprot.2006.129

[47] Takemori N., Takemori A., Tanaka Y., Endo Y., , MEERCAT: Multiplexed Efficient Cell Free Expression of Recombinant QconCATs For Large Scale Absolute Proteome Quantification*. Mol. Cell. Proteomics 2017, 16, 2169–2183. 29055021 10.1074/mcp.RA117.000284 PMC5724179

[48] Braun S.M.G., Petrova R., Tang J., Krokhotin A., , BAF subunit switching regulates chromatin accessibility to control cell cycle exit in the developing mammalian cortex. Genes Dev. 2021, 35, 335–353. 33602870 10.1101/gad.342345.120 PMC7919417

[49] Mashtalir N., D’Avino A.R., Michel B.C., Luo J., , Modular Organization and Assembly of SWI/SNF Family Chromatin Remodeling Complexes. Cell 2018, 175, 1272–1288.e20. 30343899 10.1016/j.cell.2018.09.032 PMC6791824

[50] Chambers M.C., Maclean B., Burke R., Amodei D., , A cross-platform toolkit for mass spectrometry and proteomics. Nat. Biotechnol. 2012, 30, 918–920. 23051804 10.1038/nbt.2377 PMC3471674

