# Supplementary material for: QuickProt: A bioinformatics and visualization tool for DIA and PRM mass spectrometry-based proteomics datasets
Source: bioRxiv. 2025 Mar 28:2025.03.24.645047. Preprint. [Version 1] doi: 10.1101/2025.03.24.645047 (PMC11974799; doi:10.1101/2025.03.24.645047)
Supplement: Supplement 3 [file NIHPP2025.03.24.645047v1-supplement-3.pdf]

bioRxiv preprint doi: https://doi.org/10.1101/2025.03.24.645047; this version posted March 28, 2025. The copyright holder for this preprint (which was not certified by peer review) is the author/funder, who has granted bioRxiv a license to display the preprint in perpetuity. It is made available under aCC-BY 4.0 International license.

Supplementary Methods:

Method S1: Hematopoietic stem and progenitor cell isolation, culture, and total protein extraction.

Hematopoietic stem and progenitor cell isolation was performed as previously reported [15]. Briefly, $CD34^+$ cells from umbilical cord blood donors were enriched by negative selection using the RosetteSep kit (STEMCELL Technologies; Catalog number: 15631) and the Ficoll density gradient, followed by positive selection with the Positive Selection Kit (STEMCELL Technologies, Catalog number: 18096). The purified cells were analyzed for CD34 expression via FACS (Fluorescence-activated cell sorting). $CD34^+$ cells were differentiated into the erythroid lineage using a 4-step induction protocol. First, between days 0-11, CD34+ cells grew in a serum-free IMDM (Iscove′s Modified Dulbecco′s Medium) medium supplemented with various combinations of human recombinant cytokines and other additives. Second, between days 11-15, cells were co-cultured with stromal MS-5 cells in an enriched IMDM medium supplemented with EPO (erythropoietin). Third, between days 15-18, co-culture was transferred to the enriched IMDM medium without cytokines. Fourth, between days 18-26, co-culture was incubated in an enriched IMDM medium with 10% fetal bovine serum. Cell viability and counting were conducted by FACS analysis, with samples collected on days 0, 4, 6, 8, 10, 11, 12, and 14. Two biological replicates were collected for subsequent experiments.

For total nuclear protein extraction, 10 x 10^6 cells were centrifuged at 1500 rpm for 5 minutes at 4°C. The cells were then washed with 1 mL of cold PBS (phosphate-buffered saline) pre-chilled to 4°C. The cells were suspended in 80 µL of swelling buffer (10 mM HEPES pH 8, 1.5 mM $MgCl_2$, 10 mM KCl, 0.1% NP40, 1 tablet of protease inhibitor C per 10 mL of buffer), vortexed, and kept on ice for 15 minutes with vortexing every 5 minutes. Pellet nuclei were obtained by centrifugation at 1500 rpm for 5 minutes at 4°C. The nuclei pellets were then washed twice with 1 ml of ice-cold PBS and re-suspended in 80 µL of extraction buffer 1 (50 mM HEPES pH 8, 1.0 mM $MgCl_2$, 150 mM NaCl, 1% NP40, 1 tablet of protease inhibitor C per 10 mL), vortexed 5 times for 2 seconds each, and centrifuged for 10 minutes at 13,000 rpm at 4°C. The supernatant, or nuclear extract 1 (NE1), was kept on ice or stored at -80°C. Next, 80 µL of extraction buffer 2 (50 mM HEPES pH 8, 1.0 mM $MgCl_2$, 150 mM NaCl, 0.5% (w/v) sodium deoxycholate, 1% NP40, 0.1% (w/v) SDS, 50 ng/µL benzonase, 1 tablet of protease inhibitor C per 10 mL of buffer) was added to the pellet nuclei. The mixture was vortexed for 5 minutes, shaken on a thermomixer at 14,000 rpm and 37°C for 20 minutes, vortexed again for 5 minutes, and centrifuged for 15 minutes at 13,000 rpm at 4°C. The supernatant, or nuclear extract 2

bioRxiv preprint doi: https://doi.org/10.1101/2025.03.24.645047; this version posted March 28, 2025. The copyright holder for this preprint (which was not certified by peer review) is the author/funder, who has granted bioRxiv a license to display the preprint in perpetuity. It is made available under aCC-BY 4.0 International license.

(NE2), was kept on ice or stored at -80°C. Aliquots of 3 µL were taken from NE1 and NE2 for BCA protein assay measurement (Thermo Scientific; J63283.QA), following the manufacturer's instructions. NE1 and NE2 were mixed and used for subsequent experiments.

Method S2: Production of heavily labeled QconCATs.

QconCATs (QconCAT09-12, Table S5) were cloned into the pEU-EO1-MCS expression vector (Cell Free Sciences). The coding sequences for each QconCAT are flanked by an N-terminal linker (MGASGK) and a Strep II tag (WSHPQFEK), followed by a C-terminal linker connected to a His-tag (YGGGSHHHHHHHH). Protein expression was conducted using a wheat germ cell-free protein expression system (WEPRO8240H kit; Cell Free Sciences) following the instructions of the manufacturer. During translation, isotopically heavy arginine ($^{13}C_6$,$^{15}N_4$) and lysine ($^{13}C_6$,$^{15}N_2$) are incorporated in the QconCATs. His-tagged QconCAT proteins were purified via Ni-NTA (Nitrilotriacetic acid) chromatography. Briefly, 420 µL of binding buffer (30 mM Tris pH 8.0, 500 mM NaCl, 1 mM TCEP, 8 M urea, 10 mM imidazole) were mixed with a 210 µL aliquot from the QconCAT protocol. The mixture was incubated at room temperature for 30 minutes with end-over-end rotation. Separately, the Ni-NTA beads (Thermo Fisher; Catalog number: 88222) were mixed by tilting the bottle on a longitudinal axis until homogeneous. Then, 100 µL of Ni-NTA were taken into a 1.5 mL tube and washed with ~1 mL of binding buffer. This was repeated twice, followed by centrifugation at RT, 700 g for 1 minute. The QconCAT sample was added to the washed Ni-NTA beads and incubated at RT for 60 minutes with end-over-end rotation. To equilibrate the spin column (Thermo Fisher; Catalog number: 89879), 400 µL of binding buffer was flowed through the column by centrifugation at 700xg for 1 minute at RT, and the flow-through was discarded. The sample and Ni-NTA beads were poured into the spin column inserted in a collection tube and centrifuged at 700xg, RT for 1 minute to collect the flow-through. The column was then inserted into a new collection tube and washed with 400 µL of wash buffer (30 Tris pH 8.0, 500mM NaCl, 1mM TCEP, 8 M urea, 25 Imidazole) three times. The column was moved to a new collection tube and eluted with 20 µL of elution buffer (30 mM Tris pH 8.0, 500 mM NaCl, 1 mM TCEP, 8 M urea, 250 Imidazole) 4-5 times. The eluates (His-tagged purified QconCATs) were stored at -80 °C. To assess the yield of His-tagged purified QconCATs, we used a known amount of commercially available Strep II tag peptide (GenScript) and quantified QconCAT amount via SID-PRM MS-proteomics.

Method S3: Sample processing for MS-proteomics.

The total protein of nuclear extracts was normalized to 10 µg for all erythroid samples. 2.5 ng of each heavy-labeled QconCAT (QconCAT09-12, Table S5) was spiked into the nuclear extracts. LCMS-grade water (Thermo Scientific; Catalog number: 047146.K2) was added to reach a total starting volume of 30 µL. Next, 30 µL of 2x lysis buffer (10% SDS [Sodium dodecyl sulfate]; 100 mM ABC [Ammonium Bicarbonate]) was added to the sample in safe-lock protein low-bind 1.5 mL tubes (Fisher Scientific; Catalog number: 13698794). For reduction, 2.6 µL of 120 mM TCEP (Thermo Scientific; Catalog number: 77720) was added and incubated at 37 °C for 15 minutes without shaking. For alkylation, 2.6 µL of 500 mM CAA (Thermo Scientific; Catalog number: 148410050) was added, and the sample was incubated in the dark for 20 minutes at room temperature (RT) without shaking. The denaturation process was completed

bioRxiv preprint doi: https://doi.org/10.1101/2025.03.24.645047; this version posted March 28, 2025. The copyright holder for this preprint (which was not certified by peer review) is the author/funder, who has granted bioRxiv a license to display the preprint in perpetuity. It is made available under aCC-BY 4.0 International license.

with 6.6 µL of 55% phosphoric acid, followed by vortexing for 5 minutes. Then, 430 µL of an in-house prepared S-buffer (90% Methanol; 100 mM Tris, pH 8.0) was added and vortexed for 5 minutes. The samples were then added to columns from the S-Trap micro MS sample prep kit (Protifi; Catalog number: C02-micro-80) and centrifuged at 4,000 × g for 30 seconds at RT. This process was repeated in ~100 µL increments until the entire sample volume passed through the column, with flow-through being discarded. The columns were washed four times with 150 µL of S-trap buffer via centrifugation (4,000 × g for 30 seconds at RT) and then dried by centrifugation (4,000 × g for 30 seconds at RT). The S-trap micro spin columns were placed into 2.0 mL low-binding tubes. For digestion, 25 µL of 1 µg Trypsin-LysC mix (Thermo Scientific; Catalog number: A4100) in 50 mM ABC was added to the columns and incubated at 37 °C overnight. Samples were eluted with 20 µL of 50 mM ABC, 40 µL of 0.2% formic acid (FA), and 50% acetonitrile (ACN). Each solvent was added twice and centrifuged at 4,000 × g for 30 seconds at RT. The combined eluate was dried in a speed vacuum, and the digested peptides were re-suspended in 60 µL of 0.1% FA, representing 10 µg of peptides in a final volume of 60 µL.

Method S4: DIA and PRM sample processing in Eclipse Orbitrap LC-MS/MS.

A volume of 3 µL (0.5µg of light peptides and 0.125 ng of heavy labeled peptides) was injected into Eclipse Orbitrap LC-MS/MS instrument equipped with precolumn Acclaim PepMap 100 C18 HPLC Columns (Thermo Scientific; Catalog number: 164946) and analytical column EASY-Spray HPLC Column (Thermo Scientific; Catalog number: ES900). For DIA, the LC followed a gradient of 0-85 min (4-25% ACN), 5 min (25-30% ACN), 5 min (30-80% ACN), and 10 min (80% ACN). For PRM, the LC followed a gradient of 0-5 min (2-5% ACN), 40 min (5-32% ACN), 5 min (32-60% ACN), 5 min (60-85% ACN), and additional 10 min (85% ACN). For both DIA and PRM, a flow rate of 300 nL/min was used. MS parameters for DIA and PRM are described in detail in Tables S2 and S3, respectively.

For DIA data processing in DIA-NN, we first created a reference library by uploading FASTA sequences from the UniProt human proteome. These sequences were then subjected to *in silico* digestion in DIA-NN with the following settings: 0.01 FDR, precursor range 395-1005 m/z, fragment ion range 200-2000 m/z, precursor charge range of 1-4, peptide length of 7-30, protease Trypsin/P (max cleavages of 1), and enabled N-terminal methionine excision and cysteine carbamidomethylation modifications. Next, we uploaded the LC-MS/MS files into MSConvertGUI [50] software to convert the files from RAW to mzML format. Experimental samples in mzML format were loaded into DIA-NN, along with the FASTA sequences and the predicted reference library. The final outputs generated were the report-lib.parquet.skyline.speclib, report-lib.parquet, and report.tsv files. Both report-lib.parquet.skyline.speclib and report-lib.parquet are sample-specific libraries generated by DIA-NN, whereas report.tsv contains identification and peptide-protein quantification data for the analyzed samples.

For DIA data processing in Skyline, we first imported the report-lib.parquet.skyline.speclib library with default settings. Then, LC-MS/MS samples in mzML format were imported into Skyline. Product ions were configured to have the best six transitions.

bioRxiv preprint doi: https://doi.org/10.1101/2025.03.24.645047; this version posted March 28, 2025. The copyright holder for this preprint (which was not certified by peer review) is the author/funder, who has granted bioRxiv a license to display the preprint in perpetuity. It is made available under aCC-BY 4.0 International license.

In peptide settings, we chose a peptide length of 7-30, protease Trypsin/P (max cleavages of 1), and enabled N-terminal methionine excision and cysteine carbamidomethylation modifications. In transition settings, we chose a fragment ion range of 200-2000 m/z, retention time filtering of scans within 5 minutes of MS/MS IDs, and a precursor charge range of 1-4. To assess associated proteins, we used the following settings: peptides must be unique to a single protein, find the minimal protein list that explains all peptides, and remove subset proteins. To import peak boundaries, we changed the column headings in report.tsv from File.Name, Modified.Sequence, RT.Start, and RT.Stop to FileName, PeptideModifiedSequence, MinStartTime, and MaxEndTime, respectively, as these are compatible with Skyline. Finally, DIA results were exported in a CSV report called "DIA_RESULTS.csv" for downstream analysis in QuickProt-DIA (Skyline) notebooks.

For PRM upstream processing, the transition list "IsolationList.csv" file was imported into the Skyline environment, and then LC-MS/MS samples in RAW format were imported. Next, we imported an in-house BAF library called "BAF_Prosit1.blib" into Skyline. This library was created with the inbuilt Prosit tool in Skyline choosing 27 as the collision energy value. Heavy isotope labels (^{13}C and ^{15}N) to the C_6 and N_2, and C_6 and N_4 of lysine and arginine residues, respectively, were added in the peptide settings interface. PRM results were exported in a CSV report called "PRM_RESULTS_Heavy_label.csv" for downstream analysis in QuickProt-PRM (Heavy label) notebooks.

Supplementary Figures

Figure S1: Scheme of the QuickProt tool for proteomic data mining and visualization. QuickProt comprises five modules: QuickProt-DIA, QuickProt-PRM, QuickProt-PepSeq, and QuickProt-ID Search. QuickProt-DIA consists of two notebooks or pipelines: QuickProt-DIA (DIA-NN) and QuickProt-DIA (Skyline). Additionally, QuickProt-PRM includes two notebooks: QuickProt-PRM (Heavy label) and QuickProt-PRM (Label-free). QuickProt-PepSeq features two pipelines: QuickProt-PepSeq (DIA-NN) and QuickProt-PepSeq (Skyline). Lastly, QuickProt-ID Search has a notebook of the same name.

Figure S2: Overview of the QuickProt-DIA (DIA-NN) or (Skyline) notebook data preprocessing interface.

Figure S3: Overview of the quality control, peptide and protein yields, and exploratory analysis interface in the QuickProt-DIA (DIA-NN) or (Skyline) notebooks.

Figure S4: Overview of the protein abundance analysis interface in the QuickProt-DIA (DIA-NN) or (Skyline) notebooks.

Figure S5: Overview of the enrichment analysis interface in the QuickProt-DIA (DIA-NN) or (Skyline) notebooks.

Figure S6: Overview of the data preprocessing interface in the QuickProt-PRM (Label-free) notebook.

Figure S7: Overview of the data preprocessing interface in the QuickProt-PRM (Heavy label) notebook.

bioRxiv preprint doi: https://doi.org/10.1101/2025.03.24.645047; this version posted March 28, 2025. The copyright holder for this preprint (which was not certified by peer review) is the author/funder, who has granted bioRxiv a license to display the preprint in perpetuity. It is made available under aCC-BY 4.0 International license.

Figure S8: Overview of the quality control, peptide and protein yields, and exploratory analysis interface in the QuickProt-PRM (Label-free) or (Heavy label) notebooks.

Figure S9: Overview of the protein abundance analysis interface in the QuickProt-PRM (Label-free) notebooks.

Figure S10: Overview of the protein abundance analysis interface in the QuickProt-PRM (Heavy label) notebooks.

Figure S11: Overview of QuickProt-PepSeq workflow. Outputs from DIA-NN or Skyline, coupled with QuickProt-DIA, can be imported into the QuickProt-PepSeq notebook (part of the QuickProt notebook series). A given region of interest (e.g., a protein domain) is then entered into the notebook to be mapped against the DIA data of a specific experiment. As a result, an Excel file, 'PeptideMatches.xlsx' will be generated. This file contains, in different tabs, the number of peptide matches per sample and per experimental group, as well as their respective amino acid sequences. The notebook also includes an option for plotting a bar graph showing the number of peptide matches in each experimental group.

Figure S12: Overview of QuickProt-ID Search workflow. The user inputs a list of genes of interest into QuickProt-ID. Using the Unipressed (Uniprot REST) library, the notebook has been adapted to generate a CSV table with their respective protein IDs.

Figure S13: Spearman's correlation coefficient (ρ) and data distribution plots among replicates for days, 2, 4, 6, 8, 10, 11, 12, and 14 in DIA-data.

Figure S14: Distribution of the number of peptides per protein and bar plots for shared and unique proteins in DIA data. A) The number of peptides per protein plot depicts the distribution of values as well as the median value for each experimental group. Bar plots depict the median values for B) shared and C) unique proteins. A summary table with the names and numbers of proteins is generated and stored in a subfolder called 'SHARED_UNIQUE_PROTEINS' within the 'TABLES' folder. Additionally, within the same subfolder, another folder called 'Extracted' is generated to extract and store tables listing the proteins that are shared or unique for a given comparison or experimental group.

Figure S15: Heatmaps show the relative abundances of proteins in the BAF, ISWI, NuRD, INO80, SAGA, ATAC, SRCAP, and ARTX chromatin remodeling complexes during the time course in the DIA data. Log2 normalization was applied to the abundance values for proteins from days 0 to 14. The abbreviation 'n.d.' stands for 'not detected', indicating values that were not found in the datasets.

Figure S16: Protein ranking for erythroid samples collected on days 0, 2, 4, 6, 8, 10, 11, 12, and 14 in DIA data. The Y-axis represents the log2 abundance of proteins in the proteomes of each experimental group, whereas the X-axis depicts the abundance ranking for proteins in a given proteome. The names of the maximum, median, and minimum-ranking proteins are shown in the plot.

Figure S17: Volcano plots for differential expression of D0 vs. D4, D6, D8, D10, D11, or D12 from the DIA data. The volcano plots depict a P-value ≤ 0.05 and a fold-change (FC)

bioRxiv preprint doi: https://doi.org/10.1101/2025.03.24.645047; this version posted March 28, 2025. The copyright holder for this preprint (which was not certified by peer review) is the author/funder, who has granted bioRxiv a license to display the preprint in perpetuity. It is made available under aCC-BY 4.0 International license.

threshold of 1.0. In the upper part of the plot, beside the title, a detailed description of the total number of upregulated, downregulated, and non-significantly changed proteins is shown.

Figure S18: Bar plots for protein abundance and peptide numbers for selected proteins in the DIA data of the erythroid samples. A) The abundance of ARID1A (upper panel) and SMARCC2 (lower panel) proteins is displayed in bar plots. Statistical analysis by *t*-test was performed on the datasets, using Day 0 as the reference group for comparison. Asterisks represent statistically significant differences ($P \leq 0.05$), whereas 'n.s.' indicates non-significant differences. These statistical representations were added automatically by the code in the QuickProt-DIA notebook. B) Number of peptides supporting the abundance estimate for ARID1A and SMARCC2 in each experimental group. Data represent the median ± SD of two biological replicates.

Figure S19: KEGG enrichment analysis of proteomes from individual experimental groups (A-C), and for D0 vs. D2 or D14 comparisons (D-E) in DIA data.

Figure S20: KEGG enrichment analysis of upregulated proteins in proteomes for A) D0 vs. D2 or B) D0 vs. D14 comparisons from the DIA data.

Figure S21: KEGG enrichment analysis of upregulated proteins in proteomes in A) D0 vs. D2 or B) D0 vs. D14 comparisons from the DIA data.

Figure S22: GO enrichment analysis of proteomes from individual experimental groups in DIA data. GO enrichment is classified by A) Biological Process, and B) Cellular Component.

Figure S23: Molecular function GO enrichment analysis of proteomes from individual experimental groups in the DIA data.

Figure S24: GO enrichment of proteomes from D0 vs. D2 or D14 comparisons in DIA data. GO enrichment is classified by A) Biological Process, B) Cellular Component, and C) Molecular Function as depicted in the plots.

Figure S25: GO enrichment analysis of upregulated proteins from proteomes in D0 vs. D2 or D14 comparisons from the DIA data. GO enrichment is classified by A) Biological Process, B) Cellular Component, and C) Molecular Function as depicted in the plots.

Figure S26: GO enrichment analysis of downregulated proteins from proteomes in D0 vs. D2 or D14 comparisons from the DIA data. GO enrichment is classified by A) Biological Process, B) Cellular Component, and C) Molecular Function as depicted in the plots.

Figure S27: Spearman's correlation coefficient (ρ) and data distribution plots among replicates for days, 2, 4, 6, 8, 10, 11, 12, and 14 in PRM data.

Figure S28: A) Distribution of MS2 data points across the chromatographic peak and B) correlation matrix among experimental groups from the PRM data.

Figure S29: Number of A) peptides and B) proteins, and C) distribution of number of peptides per protein in PRM data. Data represent the median ± SD of two biological replicates.

bioRxiv preprint doi: https://doi.org/10.1101/2025.03.24.645047; this version posted March 28, 2025. The copyright holder for this preprint (which was not certified by peer review) is the author/funder, who has granted bioRxiv a license to display the preprint in perpetuity. It is made available under aCC-BY 4.0 International license.

Figure S30: Protein ranking for erythroid samples collected on days 0, 2, 4, 6, 8, 10, 11, 12, and 14 in PRM data. The name of every protein in the ranking is depicted in each plot. The Y-axis represents the log2 number of protein copies per nucleus in the proteomes of each experimental group, whereas the X-axis depicts the abundance ranking for proteins in a given proteome.

Figure S31: Trend line plot for protein members of cBAF complex based on the PRM data. Trend line plot for ACTL6 (ACTL6A, and ACTL6B), BCL7 (BCL7A, BCL7B, and BCL7C), DPF (DPF1-3), SMARCA (SMARCA2, and SMARCA4), SMARCC (SMARCC1, and SMARCC2), SS18 (SS18 AND SS18L1), and SMARCE1 proteins. Data represent the median ± SD of two biological replicates.

Supplementary Tables

Table S1: List of annotated tables generated for each QuickProt notebook. A detailed description of the folder location, names of output spreadsheet tables, and their contents is provided.

Table S2: DIA method for sample processing in Eclipse Orbitrap LC-MS/MS.

Table S3: PRM method for sample processing in Eclipse Orbitrap LC-MS/MS.

Table S4: Metadata table containing the names of samples (used for ease in analysis) and their respective LC-MS/MS raw file names.

Table S5: List of peptides and QconCATs used for PRM quantification.

Table S6: Molecular weight (MW) of QconCATs.

Table S7: Protein amount (pg) per nucleus in each experimental group.

Table S8: Median number of copies per nucleus of protein members of the cBAF complex. The median copies per nucleus were calculated first among QconCATs within each replicate, then these values were averaged across two biological replicates to calculate the final copies per nucleus. The minimum and maximum values for each protein across the time course are also represented in the table.
